# A Visit to Poland

**Published:** 1981

**Authors:** John Cosh

**Affiliations:** Consultant Physician, Royal United Hospital, Bath


### A Visit to Poland

**John Cosh**
Consultant Physician, Royal United Hospital, Bath

Events in Poland have been very much in the news and in our minds in recent months. Reports of the disturbing pressures on the Polish people, and the television views of street scenes in the cities have brought back vivid memorial of my visit there in the autumn of 1977. Although only there for a week I managed to see a great deal, and was much impressed by the spirit and the hospitality of the people I met. Their endurance in the war years and afterwards is legendary, and once again today they are facing a time of great stress, deprivation and uncertainty as to the future. In the light of recent events, perhaps an account of that visit will be of interest to readers.

In 1976 a symposium on thermography was held in Bath, and it was there that I met Dr. Maria Sadowska-Wroblewska who is director of clinical rheumatology at the Institute of Rheumatology in Warsaw. In answer to my many questions about Poland she replied that I have better come and see for myself. In due course, at her request, the Polish Ministry of Health issued a formal invitation for me to spend a week there at their expense – apart from the cost of travel, which was covered by Wessex Region. It was agreed that I should visit Warsaw and Cracow, and the spa town of Ciechocinek, timing my visit so that I could travel on to Czechoslovakia where there would be a rheumatological congress at Piestany, which many Polish delegates would be attending.

The outward journey was complicated by the closure of Warsaw airport for constructional work, with the result that I had to fly to Gdansk and go on by coach to Warsaw. Services at Gdansk airport, which is considerably smaller than Lulsgate, were obviously strained by the arrival of a full jet airliner from London, but we eventually got away, sustained by the issue of some very basic rations. It was a Sunday evening and our route lay through the outskirts of Gdansk, with glimpses of the port and of the old city, which was faithfully rebuilt after extensive War damage just as much of Warsar was. I noticed crowds of still and silent people around the doors of several churches, and realised that these were overflow congregations, for the insides of the churches were already full.

The coach journey to Warsaw took 6 hours, through totally flat country, mainly farmland and forest, sparsely populated, with few towns. The centre of Warsaw, which we reached at 11 p.m. seemed by contrast, handsome, brilliant and spacious. I was given a warm welcome by my hostess and her husband and taken to the Europaiski Hotel. This overlooks Victory Square and the national war memorial – the wide open space where subsequently the Pope celebrated mass before a congregation of many thousands.

### THE INSTITUTE OF RHEUMATOLOGY

The next three days I spent mainly at the Institute, which is in effect a 400 bedded hospital in a quiet part of the city. It was built immediately after the war, on rather utilitarian lines, as a national centre in which rheumatic diseases of all types could be investigated and treated. It provides in-patient and out-patient services in adult and paediatric rheumatology, and has a department of orthopaedic surgery which carries out about 700 operations annually, including some 200 hip arthroplasties. There are physiotherapy and hydrotherapy departments, and rehabilitation is undertaken partly at the Institute and partly at a spa centre some distance from Warsaw. The pathology and immunology departments carry research programmes, and the Institute is a centre for postgraduate teaching. It acts as a focus for nationwide rheumatic services, which have recently been expanded by the appointment of rheumatologists in all of the country's 48 districts.

As in other East European countries, rheumatic fever is regarded as one of the rheumatic diseases. While rheumatic fever itself has virtually disappeared today, rheumatic heart disease continues to be investigated and treated at the Institute, so that there is also in the Institute a department of cardiac surgery complete with cardiac catheter laboratory. One feature adding to the prestige of the Institute was that the Minister of Health was a cardiac surgeon on its staff.

In the 3 days that I spent in the Institute I was able to visit all of its departments and to meet many of its staff. Many of them spoke good English and there was little difficulty in communication. On the third day I was invited to give two talks, on rheumatoid arthritis, and on thermography and its clinical applications.

The overall impression was of a first class organisation, very active in all of its departments, with an intelligent and highly competent staff. The quality of the building was on the whole good, and roughly comparable with our own hospitals of similar age. However, its technical resources in many departments were poor. Here one appreciates the support given to research in Britain by voluntary bodies such as the Arthritis and Rheumatism Council, which provides some two million pounds annually. Such bodies are of course considered unacceptable in communist controlled countries, as by their very existence they imply that the provisions of the state service are less than adequate.

The great majority of the medical staff were women, mostly married; as the custom is for a married woman to retain her maiden name and hyphenate it with her husband's name, their names at first sight seemed unpronouncably complicated. They mainly appeared to work on 8 a.m.–4 p.m. routine, which at least allowed reasonable time for domestic commitments. I learned that their salaries were poor by our standards, being below that of the most favoured manual workers such as miners. Private practice outside hospital hours did not seem to be at all common, but no doubt exists (as in Russia) especially as professional salaries are so low.

### WARSAW

Warsaw is a city of broad streets, avenues and parks, two of which are the grounds of original royal palaces, the palaces themselves remaining as museums. The main central part of the city has been rebuilt in modern style after the devastation of 1944, and this section is dominated by the tall Russian-style 'Palace of Culture', for which the Poles offer deprecatory apologies. The rebuilt opera house is without exception the biggest and most handsome modern theatre I have seen, with impressive spacious marble foyers, and I had the pleasure of being taken to a performance there. However, the feature of Warsaw which the visitor remembers most is the reconstructed old city. This area, at least a kilometre across, has been painstakingly rebuilt exactly as it was prior to the war, in the styles of the 16th–18th centuries and clearly symbolises the continuity of Polish life and tradition. This reconstruction is the more striking in that Poland is a relatively poor country with a standard of daily life for its citizens below that in Britain in the immediate post-war years. In 1977 consumer goods in the shops were of poor quality and meat was in short supply, with empty shelves in the butcher's shops ('Tell them in England that you saw no queues outside the butchers'!). The situation in 1982 is clearly even worse. I was repeatedly told 'we are 90% catholic and only 1% communist'. In the streets it was noticeable that pro-communist slogans and propaganda were far fewer than in Russian or Czechoslovakia, and portraits of Lenin and communist leaders were absent. I saw occasional groups of military style militia, which today of course are much more in evidence.

### CIECHOCINEK SPA

One of Dr. Sadowska's commitments is to provide advice as a consultant rheumatologist to a sanatorium for the treatment of rheumatic diseases at the spa town of Ciechocinek. This is about 100 miles NW of Warsaw. I was invited to go with her on one of her monthly visits. This was a two day trip, with overnight accommodation at the sanatorium, and we travelled by car.

Poland has six spa towns, each with its special role and Ciechocinek is the recommended centre for rheumatic disorders. There are springs of salt water here, which led to the organisation of the spa on traditional European lines in the 19th century. However, spa facilities and sanatorium buildings have increased markedly in the last 20 years and half a million spa visitors now come annually. It is a quiet undramatic little town with pleasant gardens and parks and no particular 'distractions'. There is a group of central spa establishments providing various forms of hydrotherapy and physiotherapy, but apparently no independent hotels. Patients and visitors stay in 'sanatoria', which offer rather frugal accommodation and for the most part therapy is given within the sanatoria, each of which has its treatment facilities and staff of therapists and visiting doctors. The medical population of the town has increased from less than 10 in the 1930's to about 100 now.

Many of the sanatoria are specifically linked to an organisation or group such as a trade union. The establishment I visited with Dr. Sadowska, called 'Budowlani', serves members of the building trades union and has room for 150 patients. They are referred from all parts of the country and both active and retired members of the union and their families are provided for. The majority of the patients I saw there did not seem particularly disabled and would probably merely have been receiving out-patient physiotherapy in Britain. The normal length of stay is 24 days, which allows for initial medical assessment and then a planned programme of 3 weeks' quite intensive treatment, with 2 or 3 treatment sessions each day. Initial dental and ophthalmological assessment is carried out and for many patients a series of dental sessions is arranged within the sanatorium during the 3 weeks stay. Accommodation for the patients is in rooms with 1, 2 or 4 beds and the food, which I sampled, was substantial and basic.

While at Ciechocinek we made a brief visit to the nearby ancient fortress city of Torun, on the lower Vistula. This was the birthplace of the astronomer Copernicus and his original home is now a museum. There is also a university in the city which has been much developed in recent years.

### MONEY

I travelled by train from Warsaw to Cracow, buying my ticket with the generous allowance of zlotys presented to me by the Ministry of Health. At Warsaw central station a surprise offer was made to me by a quick-witted young opportunist, who spotted that I was a visitor likely to have foreign currency in his pocket. He offered to take me to Cracow by car in return for my train-ticket money – and no doubt too would have backed this up by a generous offer of zlotys for the dollars I was carrying. Dr. Sadowska refused on my behalf. Such black market offers to foreign visitors are commonplace, the going rate being 5 or 6 times the official rate. But the visitor finds that not very much can be bought with zlotys, though liquor is in adequate supply (and there are disturbing signs of increased consumption of this). Imported goods can be bought only at special shops in return for hard currency – the 'Pewex' shops, to be found in major cities and hotels where foreign visitors are likely to be.

### CRACOW

My host here was a young rheumatologist, Jan Miklasinski, who took me to the hotel where I spent 3 nights. The next day I went to the Department of Rheumatology, which has some 50 in-patient beds and runs out-patient clinics in association with the teaching hospital. I lectured again here, to an audience of about 50, with efficient sentency-by-sentence translation into Polish by an orthopaedic surgeon who had just spent 3 years in England.

By contrast with Warsaw, Cracow was physically undamaged in the war and has retained its old buildings and its atmosphere of the original capital of Poland. Within the city stands the fortress-palace and cathedral of Wawel, which I saw as a privileged visitor with a special guide. Cracow is an ancient universiy centre and since the was has expanded to over 20,000 students. The medical school attracts many foreign students including a number of expatriate Poles, who return from America and Canada to study, attracted by the much lower fees. I had no opportunity, however, to see the medical school or to attempt to assess its standards.

### WIELICZKA

Not far from Cracow I was taken to the historic salt mine at Wieliczka. Here the public descend to visit galleries, museums and chapels cut from the salt deposits 50-100 metres below ground. However, I was taken with a medical guide and interpreter to a lower level where a number of large chambers are used regularly as dormitories by patients with asthma and chest diseases. These patients spend a few weeks at a time in a sanatorium at Wieliczka and 5 times a week descend the mine shaft as we did, to a depth of 200 metres, walking along the passageways to their dormitories where 50 or more sleep; the nurses' duty rooms are formed of subsidiary chambers excavated from the salt. It is maintained that the still, slightly humid and salt-impregnated atmosphere is beneficial for patients with asthma and emphysema.

### CZECHOSLOVAKIA

On my final day, Dr. Miklasinski took me some 50 miles to Katowice, in Silesia, an industrial and coal mining city that would not have seemed out of place in Lancashire. Here I made a rendezvous with some friends who had just driven down from Warsaw, and continued the journey in their car. During the afternoon we crossed the border into Slovakia at Teschen, where it was noteworthy that the ceremonies necessary for five doctors to cross from one friendly socialist country to another took 1½ hours.

The 3-day conference at Piestany was well-run and interesting. Piestany itself is the biggest spa centre in Czechoslovakia, having natural hot springs, rather hotter and more copious than those of Bath, and naturally occurring hot mud. Full use is made of these resources. Although it is a small town, of 20,000 inhabitants, Piestany has accommodation for 2,500 patients, and an annual through-put of 30,000 patients. Over half are Czech, with treatment provided through the health service, but the remainder, from a wide range of countries, are clearly a valuable source of foreign currency. Professional standards are high and accommodation good.

It is remarkable that spa treatment should flourish so extensively in East and West Europe, while in Britain it has virtually ceased. Fundamentally it must be that the spa concept 'isn't quite British'; perhaps it's still seen as something continental and slightly decadent. Nevertheless it seems probable that spa treatment will persist in limited forms in Britain, though outside the NHS.

As for Poland, repression is stronger and life is harder today than when I saw it in 1977. Martial law seems likely to continue for a long time, and the only good thing about it is that the tanks and the troops are Polish and not Russian. The influence of the church, easily perceptible in 1977 is stronger now, with a Polish pope, whose first visit was an overwhelming success, and whose second visit is planned. For ordinary citizens, deprivation of freedom of movement, of union activity, of expression and of supplies of all kinds has reached an alarming state. There may not be much that we can do about it on an international scale, but on the personal level we can support the many organisations through which we can send food and medical supplies – even medical journals. The vital support that we can give the Polish people is to let them know that they have friends in other countries who care, and who show their concern.

